# The Tonsil as a Point of Entrance for Severe General Infectious Diseases

**Published:** 1898-09

**Authors:** 


### THE TONSIL AS A POINT OF ENTRANCE FOR SEVERE GENERAL INFECTIOUS DISEASES.

Jessen, of Hamburg (*Muenchner Medical Wochenschrift*, June 7, 1898), says that diphtheria may serve as the type of infectious disease which begins at the throat. The germ of scarlet fever is also believed to enter at the same portal. It is claimed that from 70 to 80 per cent. of all cases of acute rheumatism have an angina as a prodrome.

Of other diseases whose connection with initial angina is hardly suspected by the profession at large, Jessen mentions osteomyelitis, which has been found in a number of cases to date from a streptococcic infection of the tonsil.

Jessen then relates in detail a number of cases in which various grave diseases were ushered in by angina, and where a bacillary investigation of the tonsil showed pathogenic germs. Among the diseases named are acute rheumatism, pleurisy, pneumonia, pyæmia, septicæmia.—*Medical Review of Reviews.*

Senn (*Journal American Medical Association*) concludes a very valuable contribution on "Gunshot Wounds in Military Practice," with the following summary: (Italics by editor.)

*First.* In theory and practice military surgery is equivalent in every respect to emergency practice in civil life.

*Second.* The wounded soldier is entitled to the same degree of immunity against infection as persons in civil life suffering from similar injuries.

*Third. The fate of the wounded rests in the hands of the one who applies the first dressing.*

*Fourth.* The first dressing should be as simple as possible, including an antiseptic powder composed of boracic acid four parts, salicylic acid one part, a small compress of cotton, safety-pins and a piece of gauze forty inches square.

*Fifth.* Any attempt to disinfect a wound on the battlefield is impracticable.

*Sixth.* The first dressing stations and the field hospitals are legitimate places where the work of the hospital corps and company bearers is to be revised and supplemented. All formal operations must be performed in the field hospitals, where the wounded can receive the full benefits of aseptic and antiseptic precautions.

*Seventh. Probing for bullets on the battlefield must be absolutely prohibited.*

*Eighth.* Elastic constriction for the arrest of hemorrhage must not be continued for more than four to six hours for fear of causing gangrene.

*Ninth.* The X-ray will prove a more valuable diagnostic resource than the probe in locating bullets lodged in the body.

*Tenth.* Gunshot wounds of the extremities must be treated upon the most conservative plan, the indications for primary amputation being limited to cases in which injury of the soft parts, vessels and nerves suspend or seriously threaten the nutrition of the limb below the seat of injury.

*Eleventh. Operative interference is indicated in all penetrating gunshot wounds of the skull.*

*Twelfth.* Gunshot wounds of the chest should be treated by hermetically sealing the wounds under the strictest aseptic precautions.

*Thirteenth.* Laparotomy in penetrating gunshot wounds of the abdomen is indicated in all cases where life is threatened by hemorrhage of visceral wounds and the general condition of the patient is such as to sustain the expectation that he will survive the immediate effects of the operation.—*Medical and Surgical Monitor.*

